# Accuracy of a 7-Item Patient-Reported Stand-Alone Tool for Periodontitis Screening

**DOI:** 10.3390/jcm10020287

**Published:** 2021-01-14

**Authors:** Caroline Sekundo, Tobias Bölk, Olivier Kalmus, Stefan Listl

**Affiliations:** 1 Translational Health Economics Group (THE Group), Department of Conservative Dentistry, Clinic for Oral, Dental and Maxillofacial Diseases, Heidelberg University, 69120 Heidelberg, Germany; tobias.boelk@icloud.com (T.B.); Olivier.Kalmus@med.uni-heidelberg.de (O.K.); Stefan.Listl@med.uni-heidelberg.de (S.L.); 2 Department of Dentistry—Quality and Safety of Oral Healthcare, Radboud University Medical Center, Radboud Institute for Health Sciences, 6525 Nijmegen, The Netherlands

**Keywords:** periodontitis, diabetes, patient-reported outcomes, diagnosis, risk factors

## Abstract

Periodontitis is interrelated with various other chronic diseases. Recent evidence suggests that treatment of periodontitis improves glycemic control in diabetes patients and reduces the costs of diabetes treatment. So far, however, screening for periodontitis in non-dental settings has been complicated by a lack of easily applicable and reliable screening tools which can be applied by non-dental professionals. The purpose of this study was to assess the diagnostic accuracy of a short seven-item tool developed by the German Society for Periodontology (DG PARO) to screen for periodontitis by means of patient-reported information. A total of 88 adult patients filled in the patient-reported Periodontitis Risk Score (pPRS; range: 0 points = lowest periodontitis risk; 20 points = very high periodontitis risk) questionnaire before dental check-up at Heidelberg University Hospital. Subsequent clinical assessments according to Periodontal Screening and Recording (PSR^®^) were compared with pPRS scores. The diagnostic accuracy of pPRS at different cutoff values was assessed according to sensitivity, specificity, positive, and negative predictive values, as well as Receiver-Operator-Characteristic curves, Area Under the Curve (AUC), and logistic regression analysis. According to combined specificity and sensitivity (AUC = 0.86; 95%-CI: 0.76–0.95), the diagnostic accuracy of the pPRS for detecting periodontal inflammation (PSR^®^ ≥ 3) was highest for a pPRS cutoff distinguishing between pPRS scores < 7 vs. ≥7. Patients with pPRS scores ≥ 7 had a 36.09 (95%-CI: 9.82–132.61) times higher chance of having a PSR^®^ ≥ 3 than patients with scores < 7. In conclusion, the pPRS may be considered an appropriately accurate stand-alone tool for the screening for periodontitis.

## 1. Introduction

Regular screening for periodontitis is not only important to delay dental impairments, such as tooth loss, but also of particular importance for patients with other chronic diseases that may deteriorate due to the chronic inflammation caused by periodontitis. Periodontitis has been reported to be linked with chronic diseases, particularly diabetes and cardiovascular diseases [1]. Recent evidence suggests that treatment of periodontitis improves glycemic control in diabetes patients and reduces the costs of diabetes treatment [2,3,4,5]. Changes and dysregulations of the immune response are thought to be responsible for the interrelationship [6,7]; however, the underlying mechanisms are not fully understood yet. Propositions include the continuous activation of proinflammatory cytokines [8], subsequent dysregulation of the lipid metabolism [9], the induction of oxidative stress [10], or an adaptive immune response directed against periodontal pathogens [11].

Despite many efforts to improve preventive strategies, the global-, regional-, and country-level burden of periodontitis remains high [12,13,14]. Periodontitis has been described as a “silent disease” [15], which implies that patients in early stages (including bleeding, swelling, and tooth mobility) may not seek professional care before experiencing pain associated with more advanced stages of periodontitis. At the same time, however, precisely such patients at increased periodontitis risk tend to visit the dentist less frequently than other patients [16,17].

The challenge of patients at increased risk of periodontitis rarely utilizing dental services substantiates the relevance of methods for periodontitis screening outside dental settings, which can identify characteristics associated with periodontitis, particularly in its early stages. To this end, and considering that non-dental professionals often do not have the means, time, or qualification for clinical periodontal assessments, patient-reported information has been suggested as a potentially suitable alternative [18,19]. However, to be practically usable, respective tools not only need to be accurate in terms of measurement, but they also need to be short and easy to apply. Previously reported approaches for self-reports on periodontitis often involved relatively long questionnaires [20,21], often assessed the criterion validity of periodontal sub-items (non-stand-alone) of thematically broader questionnaires [22], and, above all, have included questions on previous periodontal treatment experiences [20,21,23,24,25,26,27] or previous diagnoses provided to them by a dental professional [21,24,25,26,28,29] (e.g., “Has a Dentist or Dental Hygienist Ever Told You That You Had Periodontal or Gum Disease?’’ [28]). These questionnaires therefore fall short of identifying previously undiagnosed periodontal-treatment needs in patients rarely utilizing dental services. Hence, to improve screening for periodontitis outside dental settings, a short, easy, and user-friendly periodontal risk assessment that can be performed by non-dental medical professionals or the patient him-/herself could be of substantial relevance.

Recently, the German Society for Periodontology (DG PARO) introduced a seven-item patient-reported Periodontitis Risk Score (pPRS) based on a retrospective analysis of possible risk factors and Centers for Disease Control/American Academy of Periodontology (CDC/AAP) periodontal disease classifications [30], using epidemiological data from the Study of Health in Pomerania (SHIP-0) [31,32,33,34]. This risk score is based on a periodontitis prediction model using self-reported information previously published with a concordance-statistic of 0.84 (95% CI: 0.82; 0.86), discriminating between patients with no or mild periodontitis and those with moderate or severe periodontitis according to CDC/AAP classifications [30]. The pPRS point-scoring system was devised according to the methods described by Sullivan et al. [35]. In comparison with the model by Zhan et al. [34], the concordance-statistic of the logistic regression model using the pPRS was very similar (0.83 (95% CI: 0.83; 0.85)). The authors concluded that the pPRS allowed for a comparable estimate of periodontitis, whilst being easier to record due to the clear scoring system, and that the pPRS was thus a valid instrument. They provided a template to be used by other medical professionals [36], as well as an online and app-based version [37] to be performed by the patient.

The comparison with CDC/AAP case definitions as a widely recognized reference standard is an important first step to evaluate the tool’s ability to detect periodontitis, particularly in population-based surveillance of periodontitis prevalence. In clinical practice, however, a patient undergoing dental examination will not always receive detailed six-point periodontal probing at the initial visit. In the absence of any signs for putative periodontitis, a detailed full-mouth, six-point periodontal examination of all teeth (including clinical attachment loss, radiographic bone loss, and probing depth) in each and every patient would exceed the capacity of many healthcare systems. As a routine measure, the German statutory health insurance only compensates for the use of basic periodontal screening (such as the Periodontal Screening and Recording (PSR^®^ Sponsored by Procter & Gamble: Fairfield, OH, USA); see below), focusing on inflammatory signs, which, if present, are followed up by more detailed periodontal examination.

Initially proposed by the World Health Organization, the Community Periodontal Index of Treatment Needs (CPITN) [38], referred to later as the Community Periodontal Index [39], has been a widely used periodontal screening tool. [40] However, it has increasingly been superseded by CDC/AAP periodontal disease classifications [30]. Somewhat later, the Periodontal Screening and Recording (PSR^®^) was introduced by the American Dental Association and the American Academy of Periodontology [41]. With the exception of an asterisk code that can be used to report particularities, such as tooth mobility and furcation involvement, the PSR^®^ Index is largely identical with the CPITN [42]. The two indices use a common evaluation method based on the following three clinical indicators: bleeding on probing, calculus accumulation, and probing depth. Depending on the results of PSR^®^ screening, the patient is subsequently recommended for oral hygiene measures (in case of gingivitis) or a more detailed periodontal examination and subsequent treatment planning, if necessary. Chairside PSR^®^ screening enables the identification of previously unrecognized periodontitis in individuals without signs or symptoms but requires the patient to be present in the dental office.

In order for the pPRS to be applicable in non-dental settings, its accuracy needs to be comparable to chairside dental screening. If sufficiently accurate, the pPRS may serve as a novel, practical, and ready-to-use approach for periodontal screening, to be used by other medical professionals and patients themselves. Therefore, the aim of this study was to clinically assess the pPRS and its diagnostic accuracy in comparison to the PSR^®^. Our research questions were as follows: (i) Is the pPRS diagnostically accurate in comparison to the PSR^®^? (ii) Which pPRS cutoff value yields the highest diagnostic accuracy for the pPRS?

## 2. Materials and Methods

This study was part of the German Innovation Fund project Dent@Prevent, aiming at the enhancement of medical-dental integration [43]. This study was approved by the human subjects’ ethics board of Heidelberg University (study number: S-248/2018) and was conducted in accordance with the Helsinki Declaration of 1975, as revised in 2013. The study was designed as a prospective cross-sectional survey and subsequent clinical examination among patients seeking dental care at the dental school of Heidelberg University Hospital between October 2018 and February 2019. The required sample size was estimated at 84 participants, using a medium correlation strength between pPRS and PSR^®^ [44] (Pearson’s r = 0.3, 80% power, a = 0.05). Informed written consent was obtained from all study participants. Patients were included if they were 18 years or older, visiting for a routine dental and periodontal check-up, and if they had consented to participate in the study. This study followed the Standards for Reporting Diagnostic Accuracy (STARD) guidelines [45].

Study participants were asked to complete the self-assessed pPRS without the assistance by any of the medical staff. The pPRS questionnaire consists of seven multiple-choice items, which are presented in Table 1 (see Table S1 for original German version). A summary score between 0 and 20 was possible, with 0 points reflecting the lowest risk of periodontitis and 20 points reflecting the highest risk for periodontitis.

After the completion of the pPRS questionnaire, patients were clinically examined (first by a dental student and then by a supervising dentist), as per standard procedure, at Heidelberg University Dental School. Probing depth (PD) (measured as the distance between the gingival margin and the pocket base), bleeding on probing (presence/absence), dental plaque or calculus (presence/absence), and defective dental restorations (presence/absence) were recorded, in order to calculate the PSR^®^. We evaluated patients’ mean PSR^®^ (as measurement of mean periodontal inflammation), as well as their highest PSR^®^ code (maximum PSR^®^), which is the basis for treatment recommendations. PSR^®^ case definitions and treatment recommendations are shown in Table S2. Examinations were performed by a 4th- or 5th-year dental student, and then by the supervising academic dentist, who verified all clinical findings. The clinical documentation was double-checked by the supervising academic dentist and used as clinical comparator information for purposes of the present study. The information about each patient’s age was also extracted from the clinical records of each patient.

Characteristics of the study population were analyzed by means of descriptive statistics. Univariate linear regression analysis was calculated to predict the mean PSR based on the pPRS. The diagnostic accuracy of pPRS for detecting periodontal inflammation (PSR^®^ < 3 vs. PSR^®^ ≥ 3) was assessed for different pPRS cutoff values and according to sensitivity, specificity, positive, and negative predictive values, as well as Receiver–Operator-Characteristic (ROC) curves and Area Under the Curve (AUC). In addition, a binary logistic regression was performed to illustrate the odds with which the best-performing pPRS specification identifies periodontal inflammation. All data analyses were performed with SPSS statistics version 24.0 [46].

## 3. Results

Of 104 participants who were initially screened for eligibility, a total of 88 individuals agreed to participate in the study. The age of study participants ranged between 18 and 81 years, with a mean of 57.7 (SD: ±13.4) years. Table 2 shows a cross-tabulation of pPRS items vs. PSR^®^ codes, including information on age and gender distribution.

Over two-thirds of study participants had a maximum PSR^®^ score of 3 or 4, and there were no study participants with maximum PSR^®^ scores < 2 (given ubiquitous presence of calculus). Patients with PSR^®^ Codes 3 or 4 (suggesting periodontal inflammation) were more often older, male, former smokers, had a lower educational level, and had previously experienced gum bleeding or tooth mobility. The mean PSR^®^ was 3.1 (SD: ±0.80), the mean pPRS was 7.8 (SD: ±4.3). The PSR^®^ increased with age: The mean age was 36.3 ± 17.7 years for participants with Code 2, 54.0 ± 15.0 years for Code 3, and 60.8 ± 11.1 years for Code 4.

**Figure 1:**
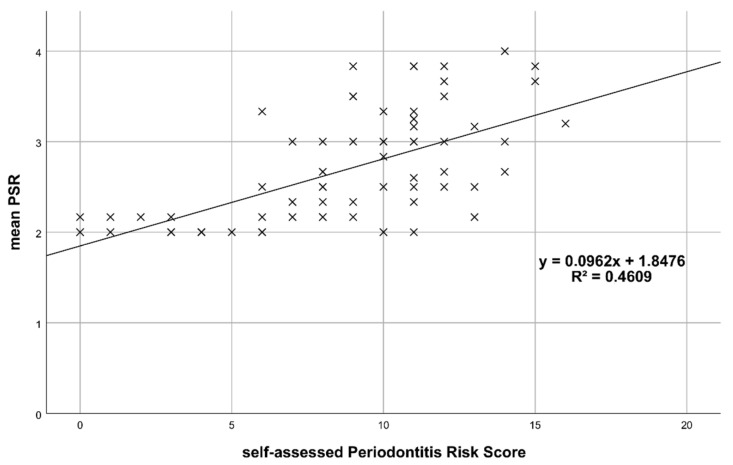
Mean PSR^®^ vs. patient-reported Periodontitis Risk Score (pPRS).

Figure 1 plots the mean PSR^®^ codes (clinically assessed) against pPRS and shows that mean PSR^®^ increases with higher pPRS. The result from the corresponding linear regression indicates that mean PSR^®^ increases significantly by 0.096 for each additional point in pPRS (*p* < 0.001; R^2^ = 0.461).

**Figure 2:**
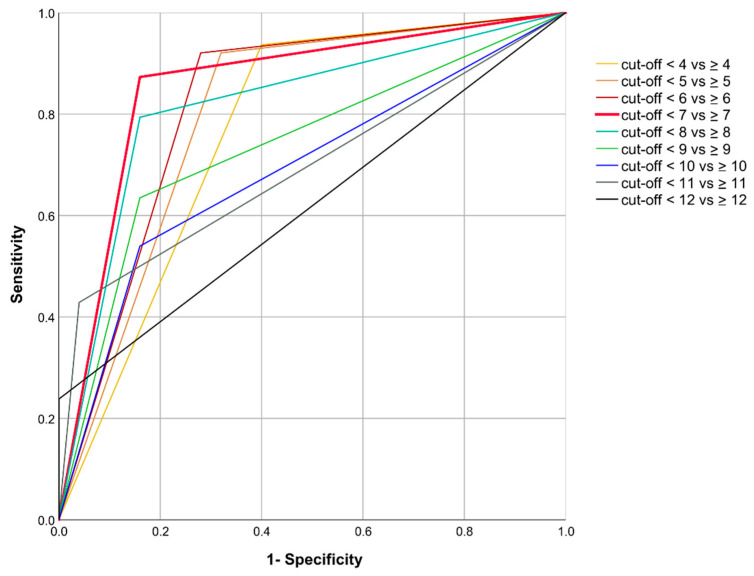
Receiver-Operator-Characteristic (ROC) curve of the patient-reported Periodontitis Risk Score (pPRS).

The diagnostic accuracy of the pPRS was assessed at various cutoff values (in the range between pPRS = 4 and pPRS = 12; please see Table S3). Sensitivity decreases and specificity increases with increasing pPRS cutoff values, with the highest reported sensitivity of 93.7% at the cutoff value of 4, and the highest reported specificity of 100% at the cutoff value of 12. According to the AUC criterion, the combined specificity and sensitivity was highest for a pPRS cutoff distinguishing between scores < 7 vs. ≥ 7 (AUC = 0.86; 95%-CI: 0.76–0.95). Corresponding ROC curves are illustrated in Figure 2. The positive predictive value for the pPRS cutoff value of 7 was 93.2%, and the negative predictive value was 72.4%.

According to logistic regression analysis (see Table 3), patients with pPRS scores ≥ 7 had a 36.09 (95%-CI: 9.82–132.61; *p* < 0.001) times higher chance of having a PSR^®^ ≥ 3 than patients with pPRS scores < 7.

## 4. Discussion

This study assesses the diagnostic accuracy of a completely stand-alone, short, and easy-to-use patient-reported screening tool for periodontitis based on patient-reported information that may be used by non-dental professionals. Our findings indicate that patients’ self-reports—measured according to the previously developed pPRS—correlate closely with the clinical examination using the PSR^®^. According to combined specificity and sensitivity, the diagnostic accuracy of the pPRS for detecting periodontal inflammation was found to be highest for a pPRS cutoff distinguishing between pPRS scores < 7 vs. ≥ 7. The results therefore suggest that the pPRS is effective in the screening for periodontitis.

The evaluated pPRS questionnaire may be particularly useful for the screening for periodontitis outside dental settings. Assessment could be performed by the patients themselves and support the patient in deciding for seeking professional oral-health advice and, if necessary, treatment. The benefits for the patient are vast. Aside from direct impacts in terms of maintaining good oral health (including avoidance of tooth loss or impaired masticatory function), the detection and management of periodontitis also contribute to avoiding detrimental impacts in terms of psychological distress and compromised quality of life [15]. If initiated early enough, periodontal treatment can warrant good oral-health-related quality of life even long after initial therapy [47]. In addition, periodontitis is a chronic disease that progresses over an individual’s lifetime; severe forms are more frequently diagnosed amongst older persons. Management of periodontitis is therefore particularly important at an older age, considering that the number of risk factors, multimorbidity, and barriers to elders’ use of dental services increase with age, and this can complicate treatment [48,49].

Given the interrelationships of periodontitis with a number of other chronic diseases, particularly diabetes and cardiovascular disease [1], the pPRS could be used by general practitioners, diabetologists, cardiologists, and others who may have a need to screen for periodontitis. In this regard, the International Diabetes Federation (IDF) has published an oral-health guideline for diabetes-care professionals [50], recommending that doctors enquire annually for symptoms of gum disease and advise patients to seek attention from a dental health professional in case of suspected disease. However, the guideline lacks validated questions that may be used by non-dental professionals. To this end, the pPRS may provide a suitable tool to complement such guidelines for the screening for periodontitis by medical professionals with a non-dental background.

The pPRS may also help facilitating better medical-dental integration, for example, as integral part of an electronic Decision Support System [51]. A more integrated management of chronic and periodontal diseases, emphasizing early disease detection, could help reducing treatment costs. Screening for periodontitis may increase the possibilities for tooth retention and reduce the risks of tooth loss and tooth replacement. Costs for tooth retention via periodontal therapy and supportive periodontal treatments have previously been shown to be relatively low compared to alternatives following tooth loss (e.g., implants or fixed partial dentures), even in periodontally impaired teeth [52,53]. Some studies also suggest the positive effects of periodontal treatment on diabetes management [54,55,56], and thus the detection of periodontitis may also increase the cost-effectiveness of both periodontitis and diabetes care [57,58]. This is particularly relevant in light of expected continuing increases in diabetes prevalence and associated treatment costs [59].

Our study has limitations. First, self-reports were compared to the CPITN/PSR^®^, which is not identical to diagnoses derived from detailed six-point periodontal examination of all teeth (including clinical attachment loss, radiographic bone loss, and probing depth) [38]. Potential disadvantages include the possible underestimation of periodontitis and the mere assessment of pocket depths without assessment of attachment loss [60,61]. Further studies comparing the pPRS to a detailed periodontal examination with subsequent calculations of the periodontal inflamed surface area (PISA) [62] and CDC/AAP classifications [30] are necessary to determine the extent to which the tool may be used to assess disease severity or the potential inflammatory burden. However, the risk of underestimating disease prevalence is particularly high in studies performing partial periodontal recording of index teeth only. This was not the case in this study, as all teeth were examined. Moreover, the PSR^®^ does not serve as a substitute for detailed periodontal examination and diagnosis (including clinical attachment loss, radiographic bone loss, and probing depth), but as a first step in the chairside screening of periodontitis. Analogously, the pPRS should only be considered a practical substitute for periodontal screening in settings other than chairside clinical dental settings.

Second, the study was carried out in one single dental academic dental center, and so the results may not be fully transferrable to other settings. This, however, does not affect the internal validity of the study, which is of primary relevance as per the purpose of the study. Study participants were recruited in a dental-care setting and may have different characteristics than patients who would be observed in non-dental settings. Individuals actively seeking dental care may be more health-conscious and better able to answer questions regarding gum bleeding or tooth mobility. The agreement between self-assessed and clinically assessed periodontal risk may therefore be somewhat different if examined within a population representative of the general public, or within a population of individuals with diabetes, that is known to visit the dentist less frequently [16,17]. In order to assess the transferability to other populations and develop best-practice solutions for use in medical offices, further studies in a non-dental environment should be performed, particularly in individuals with other risk factors related to systemic inflammation, such as diabetes mellitus, obesity, or cardiovascular disease. Nonetheless, a considerable part of the pPRS is based on information regarding age, gender, and years of education, which should be understandable irrespective of health literacy. Furthermore, the present study relied on a paper-and-pencil questionnaire. If the pPRS is intended to be used by large parts of the general population and other medical professionals, an online or app-based approach might be more appropriate and warrant further testing. Note that differences between online and paper-based delivery modes are known to be small [63,64,65].

## 5. Conclusions

Within the limitations of the present study, the pPRS was found to be an accurate and easy-to-use stand-alone tool for periodontitis screening.

## Figures and Tables

**Table 1:** Periodontitis Risk Score (Kocher et al., 2018) [31].

	Abbreviation	Question		Score
Q1.	Age	How old are you?	20–29 years old	0
			30–39 years old	2
			40–49 years old	4
			50–59 years old	6
			60–69 years old	8
			70–81 years old	10
Q2.	Gender	What is your gender?	Woman	0
			Man	1
Q3.	Current smoking	Do you currently smoke?	No	0
			Yes	2
Q4.	Past smoking	If you do not smoke at the moment, have you smoked in the past?	No	0
			Yes	1
Q5.	Education	After how many years did you finish school (including primary school)?	10 years or less	1
			More than 10 years	0
Q6.	Gum bleeding	Do your gums bleed after brushing your teeth?	No	0
			Sometimes	1
			Often	2
Q7.	Tooth mobility	Are your teeth mobile?	No	0
			Yes	3

**Table 2:** Maximum Periodontal Screening and Recording (PSR^®^) vs. items of the Periodontitis Risk Score (*n* = 88).

Periodontitis Risk Score Items		PSR^®^			
		2 *n* (%)	3 *n* (%)	4 *n* (%)	Total *n* (%)
(Q1) Age (in years)	20–29	14 (56.0)	4 (13.8)	1 (2.9)	19 (21.6)
	30–39	4 (16.0)	1 (3.4)	0 (0.0)	5 (5.7)
	40–49	1 (4.0)	2 (6.9)	3 (8.8)	6 (6.8)
	50–59	2 (8.0)	13 (44.8)	12 (35.3)	27 (30.7)
	60–69	3 (12.0)	7 (24.1)	11 (32.4)	21 (23.9)
	70–81	1 (4.0)	2 (6.9)	7 (20.6)	10 (11.4)
(Q2) Gender	Men	11 (44.0)	17 (58.6)	19 (55.8)	47 (53.4)
	Women	14 (56.0)	12 (41.4)	15 (44.1)	41 (46.6)
(Q3) Current smoking	No	21 (84.0)	27 (93.1)	28 (82.4)	76 (86.4)
	Yes	4 (16.0)	2 (6.9)	6 (17.6)	12 (13.6)
(Q4) Past smoking	No	22 (88.0)	21 (72.4)	21 (61.8)	64 (82.7)
	Yes	3 (12.0)	8 (27.6)	13 (38.2)	24 (27.3)
(Q5) (Q5) Education	≤10 years	2 (8.0)	12 (41.4)	20 (58.8)	34 (38.6)
	>10 years	23 (92.0)	17 (58.6)	14 (41.2)	54 (61.4)
(Q6) Gum bleeding	Never	20 (80.0)	19 (65.5)	20 (58.8)	59 (67.0)
	Sometimes	5 (20.0)	9 (31.0)	10 (29.4)	24 (27.3)
	Often	0 (0.0)	1 (3.4)	4 (11.8)	5 (5.7)
(Q7) Tooth mobility	No	24 (96.0)	26 (89.7)	18 (52.9)	68 (77.3)
	Yes	1 (4.0)	3 (10.3)	16 (47.1)	20 (22.7)
Total *n* (%)		25 (28.4)	29 (33.0)	34 (38.6)	88 (100)
Mean Periodontitis Risk Score (Mean + SD)		3.6 ± 3.6	7.8 ± 3.4	11.0 ± 2.2	7.8 ± 4.3

**Table 3:** Result from logistic regression analysis. (Dependent variable: PSR^®^ ≥ 3 vs. PSR^®^ < 3.).

pPRS Cutoff	Odds-Ratio (95% CI)
<7 vs. ≥7	36.09 * (9.82–132.61)

* *p* < 0.001.

## Data Availability

The data presented in this study are available on request from the corresponding author.

## Supplementary Materials

The following are available online at https://www.mdpi.com/2077-0383/10/2/287/s1. Table S1. German original version of the Periodontitis Risk Score (Kocher et al., 2018) [31]. Table S2. Main PSR® code definitions and recommended treatment. Adapted from the American Dental Association and The American Academy of Periodontology, 1992 [41]. Table S3. Diagnostic accuracy of pPRS at PSR^®^ cut-off < 3 vs. ≥ 3.
