# Supplementary material for: Accuracy of a 7-Item Patient-Reported Stand-Alone Tool for Periodontitis Screening
Source: J Clin Med. 2021 Jan 14;10(2):287. doi: 10.3390/jcm10020287 (PMC7830157; doi:10.3390/jcm10020287)
Supplement: Supplementary file 1 [file jcm-10-00287-s001.pdf]

Journal of
Clinical Medicine

MDPI

Table S1. German original version of the Periodontitis Risk Score (Kocher et al., 2018) [31].

	Risikofaktoren	Kategorien	Punkte
Q1.	Wie alt sind Sie?	20–29 Jahre	0
		30–39 Jahre	2
		40–49 Jahre	4
		50–59 Jahre	6
		60–69 Jahre	8
		70–81 Jahre	10
Q2.	Welches Geschlecht haben Sie?	weiblich	0
		männlich	1
Q3.	Rauchen Sie zur Zeit?	nein	0
		ja	2
Q4.	Wenn Sie zur Zeit nicht rauchen, haben Sie in der Vergangenheit geraucht?	nein	0
		ja	1
Q5.	Nach wie vielen Jahren haben Sie die Schule abgeschlossen?	10 Jahre oder weniger	1
		mehr als 10 Jahre	0
Q6.	Blutet Ihr Zahnfleisch nach dem Zähneputzen?	nein	0
		manchmal	1
		oft	2
Q7.	Sind Ihre Zähne beweglich?	nein	0
		ja	3

Table S2. Main PSR® code definitions and recommended treatment. Adapted from the American Dental Association and The American Academy of Periodontology, 1992 [41].

	Clinical Signs	Recommended treatment
Code 0	No pathological signs	No treatment necessary
Code 1	Bleeding on probing	Oral hygiene instructions
Code 2	Plaque, calculus and/or defective margins	Removal of calculus, correction of defective margins
Code 3	5.5mm> PD > 3.5mm	If more than two sextants receive a Code 3 score: detailed full-mouth periodontal examination and subsequent treatment
Code 4	PD ≥5.5 mm	Detailed full-mouth periodontal examination and subsequent treatment

PD = Probing depth.

Table S3. Diagnostic accuracy of pPRS at PSR® cut-off <3 vs. ≥3.

pPRS cut-off	Sensitivity % [95% CI]	Specificity % [95% CI]	PPV % [95% CI]	NPV % [95% CI]	AUC [95% CI]
<4 vs. ≥4	93.7 [85.9–98.0]	60.0 [40.5–77.5]	85.5 [76.0–92.5]	78.9 [57.6–92.9]	0.77 [0.64–0.89]
<5 vs. ≥5	92.1 [83.7–97.1]	68.0 [48.6–83.9]	87.9 [78.6–94.3]	77.3 [57.4–91.2]	0.80 [0.68–0.92]
<6 vs. ≥6	92.1 [83.7–97.1]	72.0 [52.8–86.9]	89.2 [80.2–95.2]	78.3 [59.0–91.6]	0.82 [0.71–0.93]
<7 vs. ≥7	87.3 [77.7–94.0]	84.0 [66.6–94.7]	93.2 [84.9–97.8]	72.4 [54.7–86.3]	0.86 [0.76–0.95]
<8 vs. ≥8	79.4 [68.3–88.1]	84.0 [66.6–94.7]	92.6 [83.6–97.6]	61.8 [45.0–76.8]	0.82 [0.72–0.92]
<9 vs. ≥9	63.5 [51.2–74.7]	84.0 [66.6–94.7]	90.9 [80.1–97.1]	47.7 [33.4–62.3]	0.74 [0.63–0.85]

<10 vs. ≥10	54.0<br>[41.7–65.9]	84.0<br>[66.6–94.7]	89.5<br>[77.2–96.6]	42.0<br>[29.0–55.8]	0.69<br>[0.57–0.81]
<11 vs. ≥11	42.9<br>[31.1–55.2]	96.0<br>[83.5–99.8]	96.4<br>[85.2–99.8]	40.0<br>[28.2–52.6]	0.69<br>[0.58–0.81]
<12 vs. ≥12	23.8<br>[14.5–35.2]	100<br>-	100<br>-	34.2<br>[24.0–45.5]	0.62<br>[0.50–0.74]

PPV positive predictive value, *NPV* negative predictive value; *AUC* area under the curve.
